# Pathogen reduction of monkeypox virus in plasma and whole blood using riboflavin and UV light

**DOI:** 10.1371/journal.pone.0278862

**Published:** 2023-01-20

**Authors:** Izabela K. Ragan, Lindsay M. Hartson, Elizabeth J. Sullivan, Richard A. Bowen, Raymond P. Goodrich

**Affiliations:** 1 Department of Biomedical Sciences, Colorado State University, Fort Collins, Colorado, United States of America; 2 Infectious Disease Research Center, Colorado State University, Fort Collins, Colorado, United States of America; 3 Department of Microbiology, Immunology and Pathology, Colorado State University, Fort Collins, Colorado, United States of America; U.S. Food and Drug Administration, UNITED STATES

## Abstract

**Background:**

Monkeypox virus has recently emerged from endemic foci in Africa and, since October 20, 2022, more than 73,000 human infections have been reported by the CDC from over 100 countries that historically have not reported monkeypox cases. The detection of virus in skin lesions, blood, semen, and saliva of infected patients with monkeypox infections raises the potential for disease transmission via routes that have not been previously documented, including by blood and plasma transfusions. Methods for protecting the blood supply against the threats of newly emerging disease agents exist and include Pathogen Reduction Technologies (PRT) which utilize photochemical treatment processes to inactivate pathogens in blood while preserving the integrity of plasma and cellular components. Such methods have been employed broadly for over 15 years, but effectiveness of these methods under routine use conditions against monkeypox virus has not been reported.

**Study design and methods:**

Monkeypox virus (strain USA_2003) was used to inoculate plasma and whole blood units that were then treated with riboflavin and UV light (Mirasol Pathogen Reduction Technology System, Terumo BCT, Lakewood, CO). The infectious titers of monkeypox virus in the samples before and after riboflavin + UV treatment were determined by plaque assay on Vero cells.

**Results:**

The levels of spiked virus present in whole blood and plasma samples exceeded 10^3^ infectious particles per dose, corresponding to greater than 10^5^ DNA copies per mL. Treatment of whole blood and plasma units under standard operating procedures for the Mirasol PRT System resulted in complete inactivation of infectivity to the limits of detection. This is equivalent to a reduction of ≥ 2.86 +/- 0.73 log_10_ pfu/mL of infectivity in whole blood and ≥ 3.47 +/-0.19 log_10_ pfu/mL of infectivity in plasma under standard operating conditions for those products.

**Conclusion:**

Based on this data and corresponding studies on infectivity in patients with monkeypox infections, use of Mirasol PRT would be expected to significantly reduce the risk of transfusion transmission of monkeypox.

## Introduction

Over the past 40 years, the emergence of infectious diseases in human populations has seen a steady increase [1–3]. The reasons for this increase can be associated with multiple factors, including changes in travel capabilities, regional transportation improvements, increased international trade and encroachment of human populations in areas of animal habitats [4–6]. Changes in diet and practices associated with animal husbandry and food supplies in various parts of the world only further contribute to this rate of pathogen transmission and transmission between animal and human subjects as well as the spread of these agents among human populations [7, 8]. As these transmission events continue, exposure of blood product safety to the potential of disease transmission via transfusion of blood products increases proportionally.

Screening and donor questionnaires have greatly reduced transmission rates of agents that have been identified and classified as significant transfusion risks. These include agents such as HIV, hepatitis B and C viruses, West Nile virus, bacterial contaminants, among others. The safety profile in balance and at times of equilibrium in disease outbreak is high and confidence in these products during such times also remains high. It is during times of outbreak that this equilibrium is thrown out of balance and confidence in the safety and availability of blood products among the public users of these goods and services can be shaken.

Not all emerging diseases are blood-borne or transmissible [9]. There is often a period of time, however, for those pathogens known to have associated viremia in which the unknown risk of disease transmission elevates the concern and uncertainty in the use of these products. The recent SARS-CoV-2 pandemic represented a minimal risk due to the known nature of this virus and related coronaviruses such as MERS-CoV and SARS-CoV-1 to predominantly be transmitted via respiratory secretions and their apparent lack of transfusion-associated transmission. The more recent outbreak of monkeypox virus, however, represents an entirely different situation.

Monkeypox virus is a zoonotic, re-emerging virus that is known to have an associated blood viremia in which infectious transfer of disease from donor to host has been demonstrated [10]. The virus is a double-stranded DNA virus that belongs to the genus *Orthopoxvirus*, which include smallpox (Variola) virus and vaccinia virus. Since the eradication of smallpox in 1979, smallpox vaccination was discontinued leading to waning cross-protective immunity in the older population and an increasingly naïve population that threatens human health globally. The most prevalent clinical manifestation of monkeypox virus infections in humans is a smallpox-like rash, often with vesicles, on the skin. Other clinical signs include flu-like symptoms, lymphadenopathy, muscle aches, and headache [11]. The incubation period ranges from 8 to 21 days depending on route of exposure [12]. It has been reported that most of the human-to-human transmission occurring in the current outbreak is due to sexual activity [13]. Yet other modes of transmission have been identified including respiratory, fomites, and direct contact with infected lesions [14]. Of most interest is a case report describing a nurse that was exposed via needle inoculation and tested positive by PCR for the virus in her blood prior to clinical symptoms developed. This is the first report to date demonstrating a potential hematogenous spread of the virus [15]. Although blood-borne transmission has not been demonstrated, the theoretical possibility of this agent to be transmitted by blood exists in close apposition to the known transfer by semen and body fluids generated at the site of the skin lesions [16–18]. Several recent reports call out the need to investigate the risk of transfusion transmission.

Pathogen reduction of blood products has been routinely practiced for over 15 years in many centers across the world and recently in the United States [19–22]. These methods for cellular blood components rival the use of technologies to protect the fractionated plasma component products derived from donated and renumerated blood donations in their ability to achieve inactivation of a wide range of pathogens under routine process conditions. The availability of these methods affords the community the opportunity to evaluate the potential threat of an emerging agent such as monkeypox in the context of the efficacy of such methods to reduce or eliminate infectious agents from blood donations.

We undertook these studies in order to assess the ability and capacity of one such method, Mirasol PRT, to reduce the infectious load of monkeypox virus in spiked whole blood and plasma products under routine operating conditions. The studies were guided by information related to the known titers of virus present in the blood of infected individuals and results are reported in terms of the likely impact that such reduction levels would have on the threat of disease transmission via a transfusion route.

## Materials and methods

All work with monkeypox virus was conducted in a BSL-3 laboratory setting. All human blood products used in this study were obtained via informed consent at an accredited blood banking institution (Vitalant; 717 Yosemite St., Denver, Colorado). The protocol for blood collection was approved by the WIRB Institutional Review Board (WIRB, IRB Tracking Number: 20181957). Informed consent was obtained in writing from all donors prior to donation and was witnessed by blood collection staff at Vitalant. No minors were involved in the study.

The use of these blood products at Colorado State University was reviewed by the Colorado State University Institutional Review Board which is part of the Research Integrity and Compliance Review Office (RICRO) at Colorado State University, Office of the Vice President of Research under IRB Tracking Number 19.8726H. The CSU IRB deemed the use of the products under the study protocol “to not meet the requirements of the federal definition of human subject research” due to de-identification of the products and no intended use for diagnosis or treatment of human subjects.

### Plasma products

Three plasma products were collected at an accredited blood bank and shipped overnight to Colorado State University on dry ice. The products were classified as plasma not for injection, prepared from whole blood products collected in Citrate Phosphate Dextrose (CPD) and frozen prior to shipping to ≤ -20°C.

### Whole blood products

Three non-leukoreduced whole blood (WB) products were collected in Citrate Phosphate Dextrose (CPD) anticoagulant at an accredited blood bank and shipped overnight to Colorado State University at room temperature.

### Serum samples

Human serum samples for analysis via plaque reduction neutralization test (PRNT) were collected in 2020 prior to the current monkeypox outbreak in the United States. Samples were collected at an accredited blood bank with Institutional Review Board (IRB) approval and all samples were de-identified prior to shipment to CSU. Samples were frozen following collection and shipped to CSU overnight on dry ice.

### Monkeypox virus propagation

Vero cells (CCL-81) were obtained originally from the International Reagent Resource and frozen stocks prepared. Those stocks screened negative for mycoplasma contamination and were used between total passage level 35 and 41. Virus (strain USA_2003) was amplified in Vero cell culture. Vero cells were cultured in Dulbecco’s modified Eagle’s medium (DMEM, Corning) supplemented with glucose, L-glutamine, sodium pyruvate, 10% fetal bovine serum (FBS, Peak Serum) and penicillin/streptomycin (ATCC). Inoculation of Vero cells with virus was carried out directly in DMEM containing 1% FBS. Medium harvested from infected cells 4 days after inoculation was clarified by centrifugation, supplemented with FBS to 10% and frozen to -80°C in aliquots. All virus titers were measured using a standard agarose overlay plaque assay [23]. Results are presented in number of infectious virus (plaque forming units per mL).

### Pathogen reduction process

#### Plasma

Riboflavin solution (35 mL, 500 μmol/L) was added to each product, followed by inoculation with 7 mL monkeypox virus, and the bags were placed into the Illuminator (Mirasol PRT System, Terumo BCT, Lakewood, CO) for treatment with UV light per the manufacturer’s instructions as utilized in routine practice in Europe. The device is not approved for use in The United States. The products were treated with 100% of the total recommended light dose, calculated based on the volume of each product (a full treatment consists of exposure to 6.24 J/mL UV light). Average time of treatment for these products was 6 minutes. Samples were removed from each product pre- and post-illumination for viral titer determination via plaque assay.

#### Whole blood

Each WB product was transferred to an illumination bag per the manufacturer’s instructions for treatment with UV light per the manufacturer’s instructions as utilized in routine practice in Europe. The device is not approved for use in The United States. All solution and disposables were obtained from the manufacturer (Terumo BCT, Lakewood, CO). Riboflavin solution (35 mL, 500 μmol/L) was added to each product, followed by inoculation with 10 mL monkeypox virus, and the bags were placed into the illuminator for treatment with UV light -calculated using the measured hematocrit and volume of each product—to a dose of 80 J/mL _RBC_. Average time of treatment for these samples was 53 minutes. Samples were removed from each product pre- and post-illumination for viral titer determination via plaque assay.

### Viral plaque assay

All pre- and post- illumination samples were serially diluted in sterile phosphate buffered saline (PBS). Plaque assays were performed using Vero cells at confluency in 6-well cell culture plates. Briefly, plates were washed with sterile PBS. All samples were then plated in duplicates at 100 μL per well. Plates were incubated at 37°C for 1 hour with occasional rocking. A 2 mL overlay consisting of 0.5% agarose in minimal essential media (MEM, Corning) containing 2% FBS and antibiotics was added per well and plates were incubated at 37°C up to 96 hours. The cells were fixed with 10% buffered formalin, followed by the removal of the overlay and staining with 0.2% crystal violet to visualize plaque forming units (pfu). All assays were performed in BSL-3 laboratory setting.

### Plaque reduction neutralization test

The presence of neutralizing antibodies was determined by plaque reduction neutralization test (PRNT). Briefly, serum was first heat-inactivated for 30 minutes at 56°C in a waterbath. Then serum samples were diluted two-fold in DMEM media supplemented with 1% FBS and penicillin/streptomycin starting at a 1:5 dilution on a 96-well plate. An equal volume of monkeypox virus (strain USA_2003) was added to the serum dilutions and the sample-virus mixture was gently mixed. The plates were incubated for 1-hour at 37°C. Following incubation, serum-virus mixtures were plated onto Vero plates as described for viral plaque assays. Antibody titers were recorded as the reciprocal of the highest dilution in which >50% of virus was neutralized.

### Calculation of limit of detection and log reduction

When the post-treatment samples were negative for the presence of virus plaques, the limit of detection (LOD) had been reached. All values at the limit of detection were considered less than or equal to the calculated limit of detection. The theoretical limit of detection and overall log reduction were calculated using the following equations:

$$\text{LOD}={\text{log}}_{10}\left[1/\;\left(\text{N}\;\text{x}\;\text{V}\right)\right]$$ (1)

$$\text{Log}\;\text{Reduction}={\text{log}}_{10}\;\left(\text{Starting}\;\text{Titer},\;\text{pfu}/\text{mL}\right)-{\text{log}}_{10}\;\left(\text{Final}\;\text{Titer},\;\text{pfu}/\text{mL}\right)$$ (2)

where N is the number of replicates per sample at the lowest dilution tested; V is the volume used for viral enumeration (volume inoculated/well in mL). No cytotoxicity was observed of the cell monolayer at the zero dilution, hence all replicates for determination of LOD and final titer were done at zero dilution with 6 replicates for whole blood samples and 12 replicates for plasma using 0.1 mL per replicate well.

### Statistical methods

Descriptive statistics for pathogen reduction are reported, including mean, standard deviation, and number of samples analyzed (N). Sample sizes were determined by sample and product availability and not derived from power calculations.

## Results

To determine the effectiveness of pathogen reduction technology in the face of the current global spread of monkeypox virus, we spiked units of human whole blood and plasma containing riboflavin with monkeypox virus and evaluated virus titers prior to and after UV illumination. Each bag treated received between 3.0 x 10^6^−4.45 x 10^6^ pfu/mL of virus in the whole blood products and between 2.15 x 10^6^−2.75 x 10^6^ pfu/mL in the plasma products. A slight reduction between expected titer based on dilution of stock titer and the actual titer pre-treatment was observed (Table 1). This difference of approximately 1.2–1.8 log_10_ from actual to predicted titer post-spike may be due to partial neutralization of virus by components in serum or binding of virus to components in the blood products that partially neutralizes infectivity. To address this, we tested donor serum samples collected prior to the current monkeypox outbreak by PRNT for the presence of neutralizing antibodies against monkeypox virus. No neutralizing antibodies were detected by PRNT with a 50% cutoff. Whole blood products and plasma products were treated on different days. All samples were collected and held during the processing of all replicate bags for each blood product type then plated by plaque assay at the same time. Collection and processing volumes for the whole blood and plasma products are summarized in Table 2.

**Table 1:** Stock and pre-treatment viral titers.

		Pre-treatment titers (log_10_ pfu/mL)		
**Product**	**Viral stock titer (log**_**10**_ **pfu/mL)**	**Expected**	**Measured**	**Viral loss prior to inactivation (log**_**10**_ **pfu/mL)**
Plasma	6.39	4.70	3.50	1.20
Whole Blood	6.57	4.83	3.08	1.75

**Table 2:** Blood product specifications.

	Plasma	Whole Blood
**Product volume (mL)** ^a^	301 ± 19	498 ± 9
**Hematocrit (%)** ^a^	n/a	35 ± 1
**Volume virus (mL)**	7	10
**Volume riboflavin (mL)**	35	35
**Total product volume (mL)** ^a^	338 ± 19	538 ± 9

^a^Average ± SD

**Fig 1:**
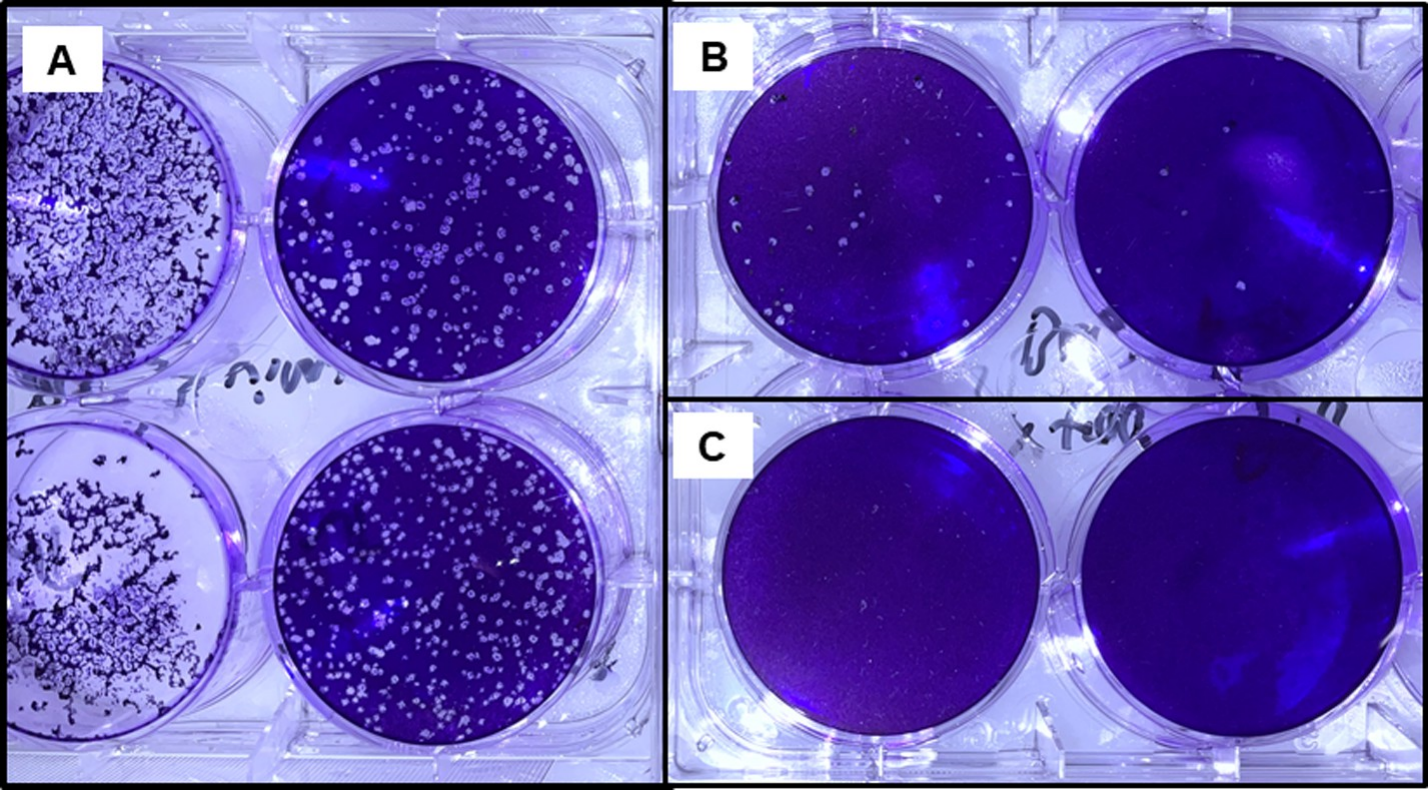
Monkeypox viral titers in whole blood products pre- and post- treatment with Mirasol. Plaque assay results from monkeypox virus pre- and post- inactivation with UV light and riboflavin. Assay performed on 6-well cell culture plates and cells stained with crystal violet for plaque visualization. A) Virus stock. B) Virus with riboflavin prior to inactivation. C) Post- inactivation.

**Fig 2:**
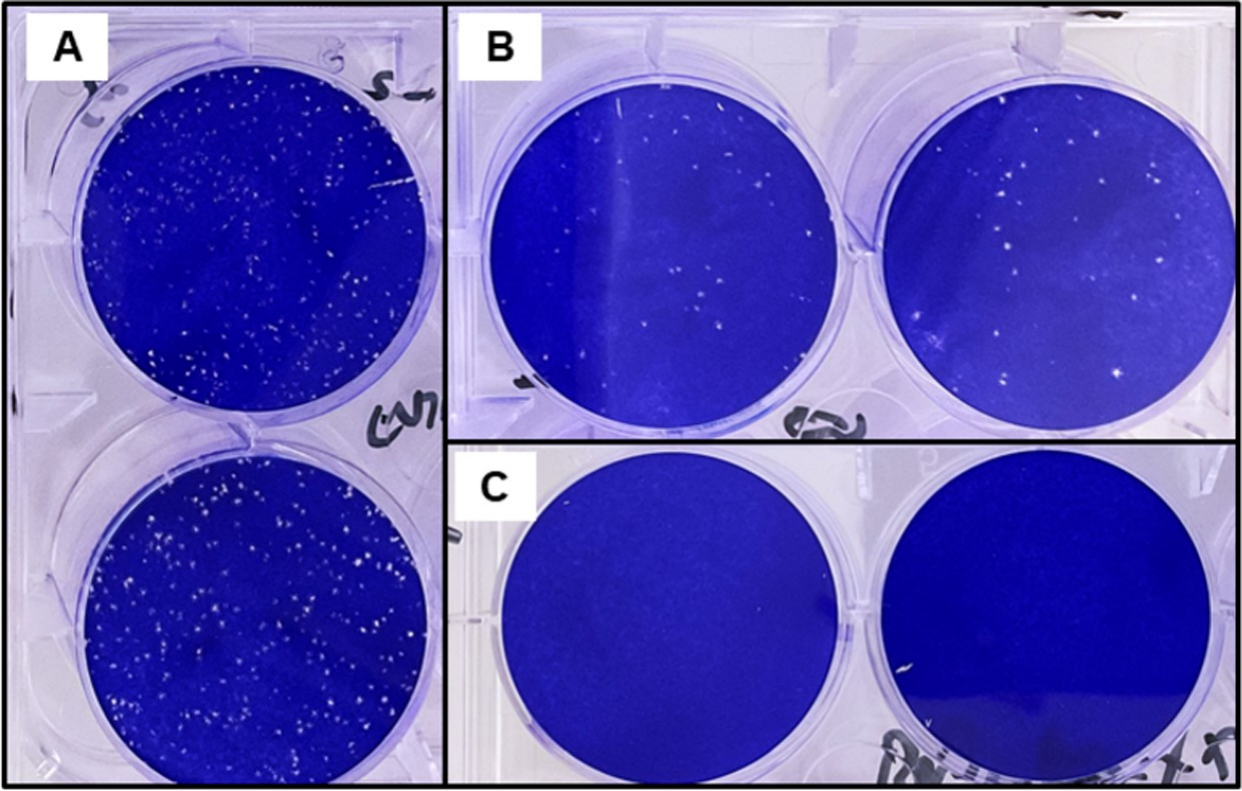
Monkeypox viral titers in plasma products pre- and post- treatment with Mirasol. Plaque assay results from monkeypox virus pre- and post- inactivation with UV light and riboflavin. Assay performed on 6-well cell culture plates and cell monolayer stained with crystal violet for plaque visualization. A) Virus stock. B) Virus with riboflavin prior to inactivation. C) Post- inactivation.

By using the plaque assay to determine infectious virus titers, we demonstrate that the UV light and riboflavin combination effectively reduced monkeypox virus in whole blood and plasma products to below the limit of detection. No viral plaques were observed after inactivation with whole blood products (Fig 1) nor with plasma products (Fig 2). This is equivalent to a reduction of ≥ 2.86 +/- 0.73 log_10_ pfu/mL in whole blood and ≥ 3.47 +/-0.19 log_10_ pfu/mL in plasma (Table 3) under standard operating conditions for those products. Based on viremia levels observed to date in human cases infected in this current monkeypox outbreak, the pathogen reduction technology should be sufficient to reduce the risk of blood transfusion via blood transfusion products [10].

**Table 3:** Viral titers pre- and post-treatment.

Product	Pre-treatment titer (log_10_ pfu/mL)	Post-treatment titer (log_10_ pfu/mL)^a^	Viral reduction (log_10_ pfu/mL)
Plasma			
1	3.42	0.00	≥ 3.42
2	3.26	0.00	≥ 3.26
3	3.64	0.00	≥ 3.64
**Average ± SD**	**3.50 ±** 0.19	**0.00**	**≥ 3.47 ± 0.19**
Whole Blood			
1	2.85	≤ 0.22	≥ 2.62
2	2.00	≤ 0.22	≥ 1.78
3	3.45	≤ 0.22	≥ 3.23
**Average ± SD**	**3.08 ± 0.73**	**≤ 0.22**	**≥ 2.86 ± 0.73**

^**a**^LODs are 0.00 log_10_ pfu/mL and 0.22 log_10_ pfu/mL for plasma and whole blood products, respectively

## Discussion

Recent studies evaluating the levels of monkeypox viremia present in patients with infection indicate levels of 10^4^ to 10^5^ copies per mL on average [10]. Importantly, these are known to be in the same range as levels found in semen, which has been demonstrated as a body fluid with potential for transmitting infection [24, 25]. Additional work by Paran et al. has demonstrated a correlation between the copy number of monkeypox virus and infectivity of those particles [26]. The values observed in patients with monkeypox virus infection correlated with 172 copy numbers per pfu and were consistent across several laboratories. The authors concluded from their work that “a total of 4,300 DNA copies per mL were required to be infectious”. Given that the levels observed in the blood of infected individuals exceeds this amount by 1–2 orders of magnitude, the likelihood that transmission of blood is possible must be considered.

The absence of a direct link between transfusion and transmission of monkeypox may be due to several factors, including the lack of hemovigilance monitoring this route of transmission, presence of potential neutralizing antibodies in many donors due to vaccination against smallpox which would reduce viral titers if not eliminate them from blood, or the possible route of administration of blood products via intravenous transfusion which may enhance immunological response mechanisms that prevent viral spread and infection. The duration of viremia in infected individuals may also be a limiting factor which reduces the potential for transmission spread. Finally, the presence of skin lesions or symptoms which may be identified at the time of blood donation can also further reduce the likelihood of disease spread via this route due to donor deferral at time of donation. The contribution of each of these factors to reducing the overall potential of disease spread via transfusion is not known at this time but does not eliminate the potential that disease spread may still occur.

Pathogen reduction methods were developed to provide a safeguard for blood products against emerging diseases, precisely at a time when disease transmission routes and frequency are unknowns but where potential exists [27]. These safeguards are meant to reduce infectious virus particles in blood while maintaining adequate protein and cellular integrity needed to support patients requiring transfusion. The extent of this protection is dependent on the viral load of the pathogen of concern and the effective range of inactivation that can be achieved by these methods under routine use conditions. The performance with individual pathogens must be evaluated as differences in structure (i.e., enveloped or non-enveloped, DNA or RNA based, etc.) may results in differences in effectiveness of these methods [28–30].

The studies performed here utilized whole blood and plasma products spiked with virus at levels of approximately 10^3^ infectious particles per mL (equivalent of approximately 10^5.2^ copies of viral DNA per mL). Viral titer did decrease after the addition to the products and prior to inactivation. We believe this may have occurred due to blood components that are binding to the virus [31]. Additionally, it is well-established that antibodies against poxviruses are cross-reactive and that the presence of antibodies in the blood products may have caused a loss of virus prior to inactivation [32, 33]. However, neutralizing antibodies against monkeypox were not detected in our plasma samples. Nonetheless, the pre-treatment titers used in this study are clinically relevant to what is being reported in patients infected with monkeypox virus. Following inactivation using the Mirasol PRT method which utilizes riboflavin and UV light, no infectious virus could be detected using a plaque assay with a limit of detection of 0 pfu per mL in plasma and 0.22 pfu per mL in whole blood. This performance and the suggested correlation between copy number and infectivity along with independent estimates of the number of copies of DNA required to produce infection suggests that this PRT method should be effective at reducing the likelihood of disease transmission via this route.

Multiple factors related to decisions about employing pathogen reduction methods in these cases are at play, including cost-benefit and risk-benefit determinations. Many locations have employed these methods in routine while others have deferred doing so either due to concerns over toxicity, product efficacy following treatment, or overall cost relative to benefit in regions where extensive screening is already taking place for the most common transfusion-transmitted diseases. Nevertheless, diseases are known to jump from animals to humans. The frequency with which this is occurring and the likelihood of further modification of disease behavior through adaptation that comes with highly plastic disease variants that multiply in humans suggests that these occurrences are likely to continue well into our future if not accelerate in pace [34]. How we address these concerns over time remains to be determined but it would seem to be prudent to factor in this known, unknown when performing such evaluations.

## Supporting information

S1 File Raw data for Table 1. (CSV)

S2 File Raw data for Table 2. (CSV)

S3 File Raw data for Table 3. (CSV)
